# Implicating *Culicoides* Biting Midges as Vectors of Schmallenberg Virus Using Semi-Quantitative RT-PCR

**DOI:** 10.1371/journal.pone.0057747

**Published:** 2013-03-08

**Authors:** Eva Veronesi, Mark Henstock, Simon Gubbins, Carrie Batten, Robyn Manley, James Barber, Bernd Hoffmann, Martin Beer, Houssam Attoui, Peter Paul Clement Mertens, Simon Carpenter

**Affiliations:** 1 VVD Program, The Pirbright Institute, Pirbright, Surrey, United Kingdom; 2 Institute of Diagnostic Virology, Friedrich-Loeffler-Institut, Greifswald-Insel Riems, Germany; Kansas State University, United States of America

## Abstract

**Background:**

The recent unprecedented emergence of arboviruses transmitted by *Culicoides* biting midges in northern Europe has necessitated the development of techniques to differentiate competent vector species. At present these techniques are entirely reliant upon interpretation of semi-quantitative RT-PCR (sqPCR) data in the form of C_q_ values used to infer the presence of viral RNA in samples.

**Methodology/Principal Findings:**

This study investigates the advantages and limitations of sqPCR in this role by comparing infection and dissemination rates of Schmallenberg virus (SBV) in two colony lines of *Culicoides*. Through the use of these behaviorally malleable lines we provide tools for demarcating arbovirus infection and dissemination rates in *Culicoides* which to date have prevented clear implication of primary vector species in northern Europe. The study demonstrates biological transmission of SBV in an arthropod vector, supporting the conclusions from field-caught *Culicoides* and provides a general framework for future assessment of vector competence of *Culicoides* for arboviruses using sqPCR.

**Conclusions/Significance:**

When adopting novel diagnostic technologies, correctly implicating vectors of arboviral pathogens requires a coherent laboratory framework to fully understand the implications of results produced in the field. This study illustrates these difficulties and provides a full examination of sqPCR in this role for the *Culicoides*-arbovirus system.

## Introduction

Schmallenberg virus (SBV) is a pathogen of ruminants which was initially identified in late 2011 through metagenomic studies conducted by the Friedrich Loeffler Institute in Germany [1]. Infection with SBV in adult sheep and cattle can result in a mild disease whose clinical signs include diarrhea and reduced milk yield. A major economic impact of SBV infection, however, lies in the occurrence of congenital defects in offspring of infected ruminants [1], [2]. Recent phylogenetic analyses have demonstrated that SBV is most closely related to viruses of the species Sathuperi virus and is not a reassortant with other species of the Simbu serogroup [1], [3]. Viruses of this serogroup, including Akabane (AKAV) and Aino virus, have previously been isolated from pools of livestock associated *Culicoides* biting midges in Japan where the vector most clearly implicated is *Culicoides oxystoma* [4], [5], [6]. Vector competence studies for these arboviruses are most complete for AKAV, particularly in Australia where the role of *Culicoides brevitarsis* in transmitting and spreading the virus has been characterized in detail [7], [8]. Incidence of AKAV in field collected *Culicoides* has also been studied in Israel [9], [10], the Oman [11] and the Republic of South Africa [12], where the major Afro-tropical arbovirus vector *Culicoides imicola* is believed to play a primary role in transmission.

Prior to the incursion of SBV, *Culicoides* biting midges were implicated as vectors of arboviruses in northern Europe during outbreaks of bluetongue virus (BTV) from 2006–2009. This process was complicated by a lack of understanding of the complexity of arbovirus infection in *Culicoides*, combined with an increasing reliance on semi-quantitative real-time RT-PCR (sqPCR) [13], [14], replacing traditional virus isolation. A major challenge in interpreting arbovirus cycle threshold (C_q_) values derived by sqPCR from pools of *Culicoides* lies in the fact that sub-transmissible infections are common in *Culicoides* occurring most frequently during initial infection and release from the hind mid-gut [15], [16]. This renders the numerous studies where only small numbers of positive pools of *Culicoides* are reported difficult to interpret [17], [18], [19], [20], [21], [22], although comparison with predicted quantities of virus in the original blood meal in larger studies can be used to demonstrate that at least limited replication of virus has occurred in the putative vector and that wide-scale infection of *Culicoides* did occur [23].

Following the incursion of SBV, several studies have attempted to address the issue of potentially confounding sub-transmissible infections by attempting to detect viral RNA in the heads of field collected *Culicoides* [24], [25]. These rely on earlier studies that demonstrated that *Culicoides* lack salivary gland barriers to infection with BTV, inferred from the fact that intrathoracic inoculation of the virus results consistently in *Culicoides* possessing fully disseminated infections [26], [27]. Hence, if replicating BTV is detected in the head there is no known subsequent barrier to full dissemination in the *Culicoides*. While a clear improvement on processing whole *Culicoides*, a key difficulty in interpreting these studies is the potential for contamination of samples with traces of SBV RNA from the original blood meal. In addition, the studies also rely on the unproven assumption that salivary gland barriers do not exist in *Culicoides* for SBV.

In this study we assess an sqPCR assay for use in detecting replicating and disseminating SBV in colony populations of two *Culicoides* species: *C. nubeculosus* and *C. sonorensis*. *C*. *nubeculosus* is found across the Palaearctic region and has been found to be refractory for arbovirus infection in a series of laboratory studies [15]. *C*. *sonorensis* is a major vector of BTV in the USA and the colony line used here has previously been shown to be competent for an AKAV strain [28]. By comparing and contrasting the replication of SBV in these two species using two different methods of infection, we have developed methods that can be applied to detection of emerging arboviruses in field-collected *Culicoides* and provide convincing evidence that this genus can act as biological vectors of SBV.

## Materials and Methods

The SBV strain used was derived from an isolation made using a *C. sonorensis* embryonic cell line in Germany by the Friedrich Loeffler Institut (FLI) and then sent to The Pirbright Institute following a single passage in a baby hamster kidney-21 (BHK-21) cells. At The Pirbright Institute the virus was then passaged twice on a BHK-21 cell line for intrathoracic (IT) inoculation studies and then passaged again on the same cell line prior to use in oral infection of *C. sonorensis* and *C. nubeculosus*. All viruses were used at C_q_ values of 10–12 with infectivity on BHK-21 cells recorded as 5.0–5.5 log_10_TCID_50_. *C*. *sonorensis* used were of the PIRB-s-3 strain, originally derived from the Sorona (AA) line propagated in Denver, Colorado, USA [29] while the *C. nubeculosus* line originated from UK specimens. Both lines have been maintained at The Pirbright Institute since the late 1960’s using standardized techniques [30].

### Intrathoracic Inoculation of *C. sonorensis*

Approximately 300 *C. sonorensis* were lightly anaesthetized with CO_2_ and then IT inoculated with 0.2 µl of SBV using pulled glass capillary needles (Narishige, Japan) and a micro-injector equipped with a foot driver (Drummond Scientific Nanoject II: Drummond Scientific, USA). Ten IT inoculated *C. sonorensis* were processed immediately by sqPCR and the remainder incubated for 10 days at 25±1°C with access to 10% sucrose solution. At day 10, surviving *C*. *sonorensis* were exposed in two groups to FTA® cards baited with Manuka honey using a previously described technique for detection of arboviruses in the saliva of mosquitoes [31]. This technique relies upon detecting traces of virus elicited during sugar feeding from individuals possessing fully disseminated infections on the FTA® cards. *C. sonorensis* were then immobilized using CO_2_ and fixed to a piece of masking tape with their ventral surface exposed. A drop of pillocarpine (parasympathomimetic alkaloid: Sigma Aldrich, UK) solution was then applied to the ventral surface of each *C. sonorensis* and saliva collected into a 1 µl microcapillary glass tube containing 10% FBS Glasgow’s media [32]. The collected media was then expelled into individual eppendorf tubes containing 0.5 ml of Schneider’s *Drosophila* Media (Gibco™) containing 10% fetal bovine serum (SDM). These final solutions were then stored at +4°C prior to analysis.

The ten *C. sonorensis* used for saliva recovery were subsequently decapitated using sterile needles (Monoject™ hypodermic needle, 18 g×1.5: Covidien, USA). Heads were ground in 100 µl of SIM containing 1000 IU/ml Penicillin/Streptomycin and 4 µg/ml Amphotericin B using two coverslips. The remaining abdomen and thorax of each individual were also homogenized for 1 min at 25 hz in 100 µl of SIM using a TissueLyser® (Qiagen, UK) and 3 mm stainless steel beads (Dejay Distribution Ltd., UK) [33]. In addition, a further 38 surviving *C. sonorensis* were homogenized as whole insects in 100 µl of SDM using the TissueLyser® system.

### Oral Infection of *C. sonorensis* and *C. nubeculosus*

Batches of approximately 300–400, 2–3 day old adult *C. sonorensis* and *C. nubeculosus* were allowed to feed on a defibrinated sheep-blood (TCS Biosciences, UK)/SBV suspension via the Hemotek system (Hemotek Ltd, UK), using a Parafilm® membrane (Cole-Parmer, UK). Ten membrane fed *C. sonorensis* and *C. nubeculosus* were processed immediately for SBV RNA and the remainder incubated for 10 days at 25±1°C with access to 10% sucrose solution. Following incubation, 19 *C. sonorensis* and 20 *C. nubeculosus* were dissected as previously described for IT inoculated individuals. A further 304 *C. sonorensis* and 150 *C. nubeculosus* were also homogenized as whole insects. As an additional assessment of the presence of infectious SBV in incubated *C. sonorensis*, 30 individuals were fed and then selected following 10 days incubation at 25°C, dissected and processed as for IT inoculated individuals. Homogenates of heads and abdomen/thorax from individuals with what were thought to be fully disseminated infections were inoculated onto BHK-21 monolayers in 25 cm^2^ flasks containing 10 ml of SDM and assessed by observation of cytopathic effect at days 1,2,3 and 4 post-inoculation. RNA was quantified in flasks immediately following inoculation using sqPCR and then at 4 days post-inoculation. Replication was assessed from the appearance of cytopathic effect in samples and by comparison of the initial and final sqPCR C_q_ values. Confirmation of virus presence in the saliva of orally infected *C. sonorensis* at day 10 post infection was also assessed as described above for intrathoracic infected *Culicoides*. Four groups of approximately 70 orally infected *C. sonorensis* each were allowed to feed on FTA® cards baited with Manuka honey and card processed as previously described [31].

### Detection of Schmallenberg Virus

Nucleic acid extraction was carried out using a Universal Biorobot (Qiagen, UK) in a 96-well format using a QIAamp® All Nucleic Acid MDx Kit (Qiagen, UK). SBV RNA in *Culicoides* samples was quantified using a sqPCR devised by the FLI that targeted the S segment of the genome [1], [34]. Duplicate assays were conducted from each extraction for the *C. sonorensis* studies only. In addition, infectious virus was isolated and quantified from selected samples using serial dilution and blind passage on BHK-21 cells. Presence of infectious virus was subsequently confirmed using observation of cytopathic effect at days 3 and 5 post-inoculation and by the sqPCR assay.

### Statistical Methods

To compare C_q_ values of dissected, IT inoculated *C. sonorensis* a linear mixed model was used with C_q_ value as the dependent variable, body component (abdomen/thorax, head or saliva) as a fixed effect and individual as a random effect. The methods were implemented using the nlme package [35] in R [36].

The C_q_ values obtained when *Culicoides* were infected by membrane feeding and processed as whole insects were analysed using a two-component mixture model [37]. In this approach we assume that the C_q_ values for *Culicoides* with transmissible infections are drawn from one distribution, while those with sub-transmissible infections are drawn from another distribution. Based on these distributions we can assign each *Culicoides* to either the “transmissible” or “sub-transmissible” group with a certain probability based on its C_q_ value and, hence, estimate the proportion of *Culicoides* with a transmissible infection. Importantly, this avoids the need to use a potentially arbitrary threshold to define transmissible and sub-transmissible infections.

More formally, we assume the C_q_ values for transmissible and sub-transmissible infections are drawn from normal distributions with different means and standard deviations. In this case, the normally-distributed C_q_ value for a *Culicoides* is conditional on its Bernoulli-distributed (and unobserved) infection status, so that,
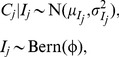
(1)where *C_j_* is the observed C_q_ value for the *j*th *Culicoides*, *I_j_* is the (unobserved) status of *Culicoides j* (i.e. transmissible (*I_j_* = 1) or sub-transmissible (*I_j_* = 0) infection), µ*_i_* and σ*_i_* are the mean and standard deviation of the C_q_ value for *Culicoides* of status *i*, respectively, and ϕ is the probability of developing a transmissible infection (i.e. competence). From this, we can use Bayes’s Rule to compute the probability that a *Culicoides* has a transmissible infection given its C_q_ value, so that,
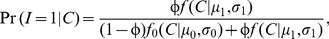
(2)where f is the probability density function (PDF) for the normal distribution and f_0_ is the PDF for the normal distribution “zero-inflated” to incorporate observations with no C_q_ value, so that
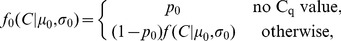
(3)where p_0_ is the probability of no C_q_ value.

The mixture model was implemented in a Bayesian framework, which requires a likelihood function and a joint prior distribution for the parameters. For the two-component mixture model, (1), the likelihood for the data is,
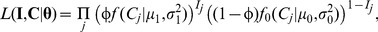
(4)where *f* is the probability density function (PDF) for the normal distribution (with mean µ*_i_* and standard deviation σ*_i_*), **θ** = {ϕ,μ_0_,μ_1_,σ_0_,σ_1_,*p*_0_} is a vector of parameters, **I** is a vector indicating the (unobserved) status of each *Culicoides* and **C** is a vector of observed C_q_ values. To ensure that the parameters in the model are identifiable, the mean C_q_ values for *Culicoides* with transmissible infections was constrained to be lower than the mean for *Culicoides* with sub-transmissible infections (i.e. µ_1_<µ_0_).

Non-informative priors were used for all parameters: Uniform (0,1) or diffuse exponential with mean 100, as appropriate. The only exception was the mean and standard deviation for C_q_ values in *Culicoides* in with a transmissible infection (μ_1_ and σ_1_) for *C. nubeculosus*, where informative priors were necessary for the methods to converge. Priors for these two parameters were constructed using the data on C_q_ values in *Culicoides* infected via membrane feeding and tested on day 0, which were assumed to reflect the C_q_ values that would be observed in *Culicoides* with a transmissible infection. A normal prior was used for μ_1_ with mean equal to the estimated mean (24.24) and standard deviation (0.70) chosen so that 50% of the prior covered the 95% confidence interval. An exponential prior was used for σ_1_ with mean equal to the estimated standard deviation (0.66). The priors were assumed to be independent of one another.

Parameter estimation was implemented in OpenBUGS (version 3.2.2; www.openbugs.info); the OpenBUGS code is supplied in the supporting information (Text S1). Two chains, each of 200,000 iterations, were run, with the first 50,000 iterations discarded to allow for burn-in of the chain. The chains were then thinned (taking every twentieth iteration) to reduce autocorrelation amongst the samples. Convergence of the MCMC scheme was assessed visually and using the Gelman-Rubin statistic in OpenBUGS.

Posterior predictive checking was used to assess model fit [37]. More specifically, the posterior predictive distribution was used to generate replicated data by sampling parameter sets from the joint posterior distribution and using the sampled parameters to simulate data-sets using the model for the C_q_ values, (1). If the observed data generate a more extreme value of the measures than the replicate data (i.e. lie outside the 95% prediction interval), this provides an indication that the model does not adequately capture the data. In this case, histograms of the observed and simulated data were compared (with simulated C_q_ values above the maximum observed C_q_ value classified as giving no C_q_ value). In addition, the posterior predictive distribution was used to explore whether or not the probability of having a transmissible infection and the distribution of C_q_ values in *Culicoides* with a transmissible infection inferred from processing whole insects were consistent with the results for dissected insects, both intrathoracically inoculated and orally infected (i.e. lie within the 95% prediction interval).

## Results

### Dissemination of SBV in *C. sonorensis* and *C. nubeculosus*

**Figure 1:**
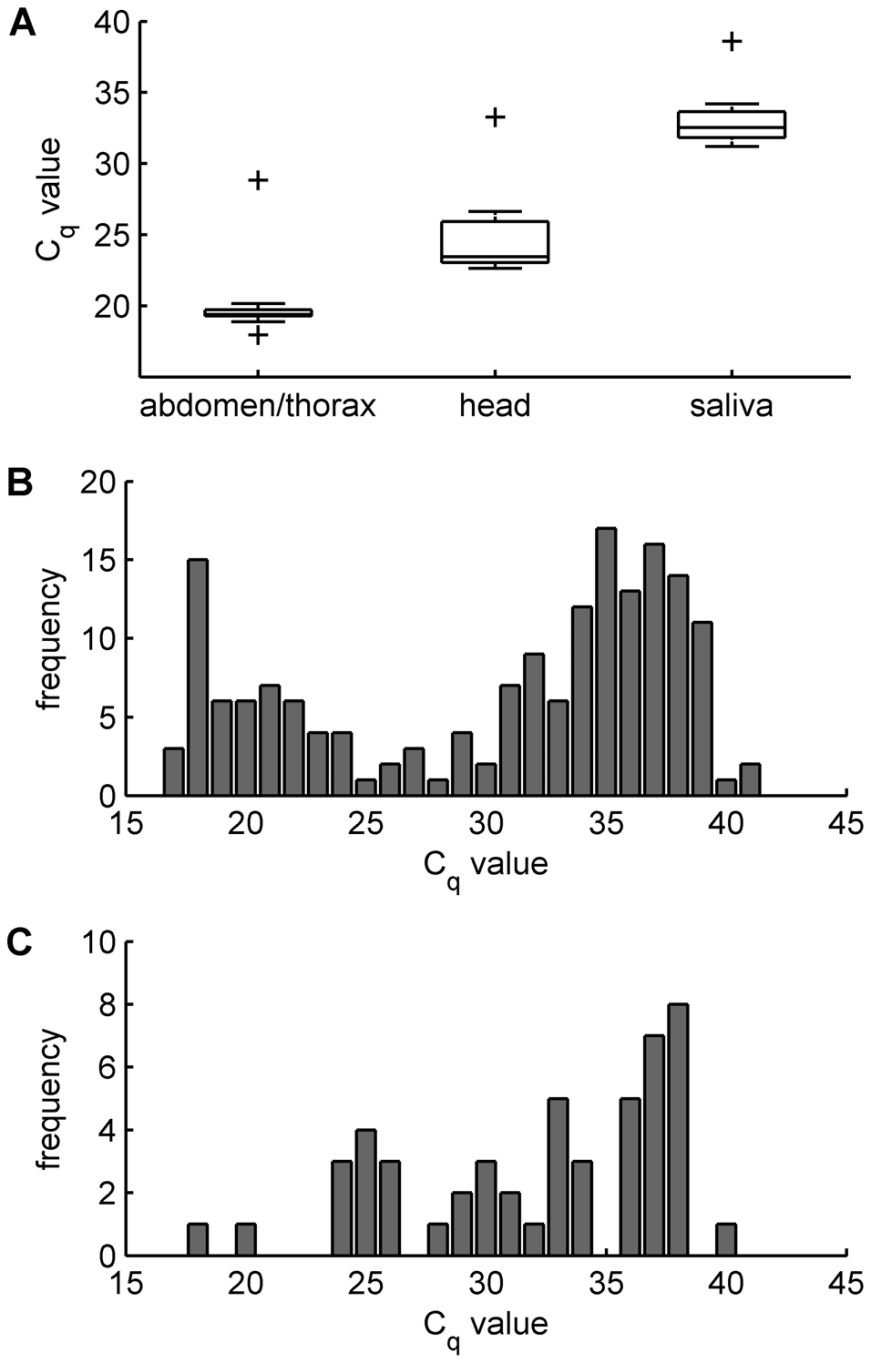
Observed C_q_ values for Schmallenberg virus (SBV) in *Culicoides* biting midges infected via different routes and processed ten days post infection. (A) *C. sonorensis* infected via intrathoracic inoculation, incubated for ten days after which the abdomen/thorax, head and saliva of individual insects were processed separately using sqPCR. The box-and-whisker plot shows the median (horizontal line), interquartile range (box), 1.5 times the interquartile range (whiskers) and any outliers (crosses). (B) *C. sonorensis* fed orally on SBV-infected blood via a membrane-based system and processed separately as whole insects using sqPCR (C) *C. nubeculosus* fed orally on SBV-infected blood via a membrane-based system and processed separately as whole insects using sqPCR.

IT inoculation led to fully disseminated SBV infections in all (10 out of 10) *C. sonorensis* examined (characterized by recovery of SBV RNA from the abdomen/thorax and the head). Recovery of SBV RNA from saliva was less consistent, with 8 (out of 10) individuals producing a positive response. The linear mixed model indicated there were significant (*P*<0.001) differences in C_q_ values between the dissected body parts and saliva, with abdomen/thorax having the lowest C_q_ value, followed by the head and the highest C_q_ values in saliva (Table 1, Figure 1A). Of the dissected, orally infected insects, one (out of 19) *C. sonorensis* and one (out of 20) *C. nubeculosus* contained a fully disseminated infection. In *C. sonorensis* the C_q_ values (duplicate samples) for the abdomen/thorax, head and saliva were 18.98/19.20, 21.29/21.76 and 31.63/31.93, respectively. In *C. nubeculosus*, the C_q_ values (single samples) were 18.20, 21.52 and 34.24, respectively. The FTA® card technique yielded at least one positive C_q_ value for both groups (of approximately 20 individuals each) of IT inoculated *C. sonorensis* (card 1∶34.21/34.73; card 2∶35.82/no C_q_). In orally infected *C. sonorensis*, all four groups of approximately 70 *C. sonorensis* tested also produced repeatable positive C_q_ values using this technique (FTA® card 1∶34.18/34.23; card 2∶35.34/35.49; card 3∶36.46/35.52; and card 4∶36.47/38.46).

**Table 1:** Differences in C_q_ values between the dissected body parts and saliva for *C. sonorensis* infected by intrathoracic inoculation with SBV.

Parameter	Estimate†	95% confidence interval
intercept	20.25	(18.46–22.04)
body part		
abdomen/thorax	baseline	–
head	4.64	(3.89–5.38)
saliva	12.79	(11.96–13.62)
standard deviation of random effect	2.76	

† coefficients in the linear mixed model for C_q_ values.

### Detection of SBV in *C. sonorensis* and *C. nubeculosus* Processed as Whole Insects

For the *C. sonorensis* processed as whole insects the observed C_q_ values showed a clear bimodal distribution with one peak at a C_q_ value of around 20 and the other at a C_q_ value of around 35 (Figure 1B). Fitting the mixture model, (1), to these data the probability of developing a transmissible infection (i.e. competence) was estimated to be 18.7% (95% credible interval 14.3–23.3%) (Table 2; Figure S1). The mixture model provided an acceptable fit to the observed C_q_ values (Figure S2A). Furthermore, the results of the mixture model (based on whole-insect processing) are comparable with the results for dissected *Culicoides* (both intrathoracically inoculated and orally infected). In particular, the number of *Culicoides* orally infected which develop a fully disseminated infection is consistent with probability of developing a transmissible infection estimated from the mixture model (Figure S3A). Similarly, the C_q_ values observed in the abdomen/thorax and the head for those *Culicoides* infected via either route with fully disseminated infections are within the range expected for *Culicoides* with a transmissible infection (Figure S3B).

**Table 2:** Estimates for vector competence and the mean and standard deviation for C_q_ values in *Culicoides* biting midges with transmissible and sub-transmissible infections after feeding on SBV-infected blood via a membrane.

Parameter	mean†	median†	95% credible interval†
*C. sonorensis*			
probability of developing a transmissible infection (φ)	0.19	0.19	(0.14–0.23)
mean C_q_ value			
transmissible infection (µ_1_)	21.05	21.05	(20.28–21.83)
sub-transmissible infection (µ_0_)	35.72	35.73	(35.14–36.28)
standard deviation of C_q_ value			
transmissible infection (σ_1_)	2.70	2.69	(2.13–3.22)
sub-transmissible infection (σ_0_)	2.96	2.94	(2.56–3.46)
Probability of no C_q_ value in a sub-transmissible infection (*p*_0_)	0.53	0.53	(0.47–0.60)
*C. nubeculosus* ‡			
probability of developing a transmissible infection (φ)	0.07	0.07	(7.5×10^−4^–0.15)
mean C_q_ value			
transmissible infection (µ_1_)	24.53	24.54	(23.34–25.78)
sub-transmissible infection (µ_0_)	34.48	34.94	(31.51–36.53)
standard deviation of C_q_ value			
transmissible infection (σ_1_)	1.62	1.93	(0.03–3.41)
sub-transmissible infection (σ_0_)	4.02	3.47	(2.27–6.49)
probability of no C_q_ value in a sub-transmissible infection (*p*_0_)	0.71	0.71	(0.61–0.79)

† summary statistics for the marginal posterior distributions (see Figure S1);

‡ the posterior distribution for *C. nubeculosus* is bimodal (Figure S1) and the summary statistics must be treated with caution.

The results for *C. nubeculosus* were more equivocal and, in particular, there was no clear bimodal distribution in C_q_ values for individuals processed as whole insects (Figure 1C). This was reflected in the parameter estimates for the mixture model (1), where the posterior distribution was bimodal (Table 2; Figure S1). Although the probability of developing a transmissible infection could not be estimated with any great precision, it is possible to conclude that it is significantly (*P*<0.001) lower than that for *C. sonorensis*. The mixture model provided an acceptable fit to the data (Figure S2B). In addition, the number of *Culicoides* orally infected which develop a fully disseminated infection is consistent with probability of developing a transmissible infection predicted by the mixture model (Figure S3C). The C_q_ values predicted by the mixture model, however, are higher than for that observed in the abdomen/thorax and the head for the one *Culicoides* with a fully disseminated infection (Figure S3D).

**Figure 2:**
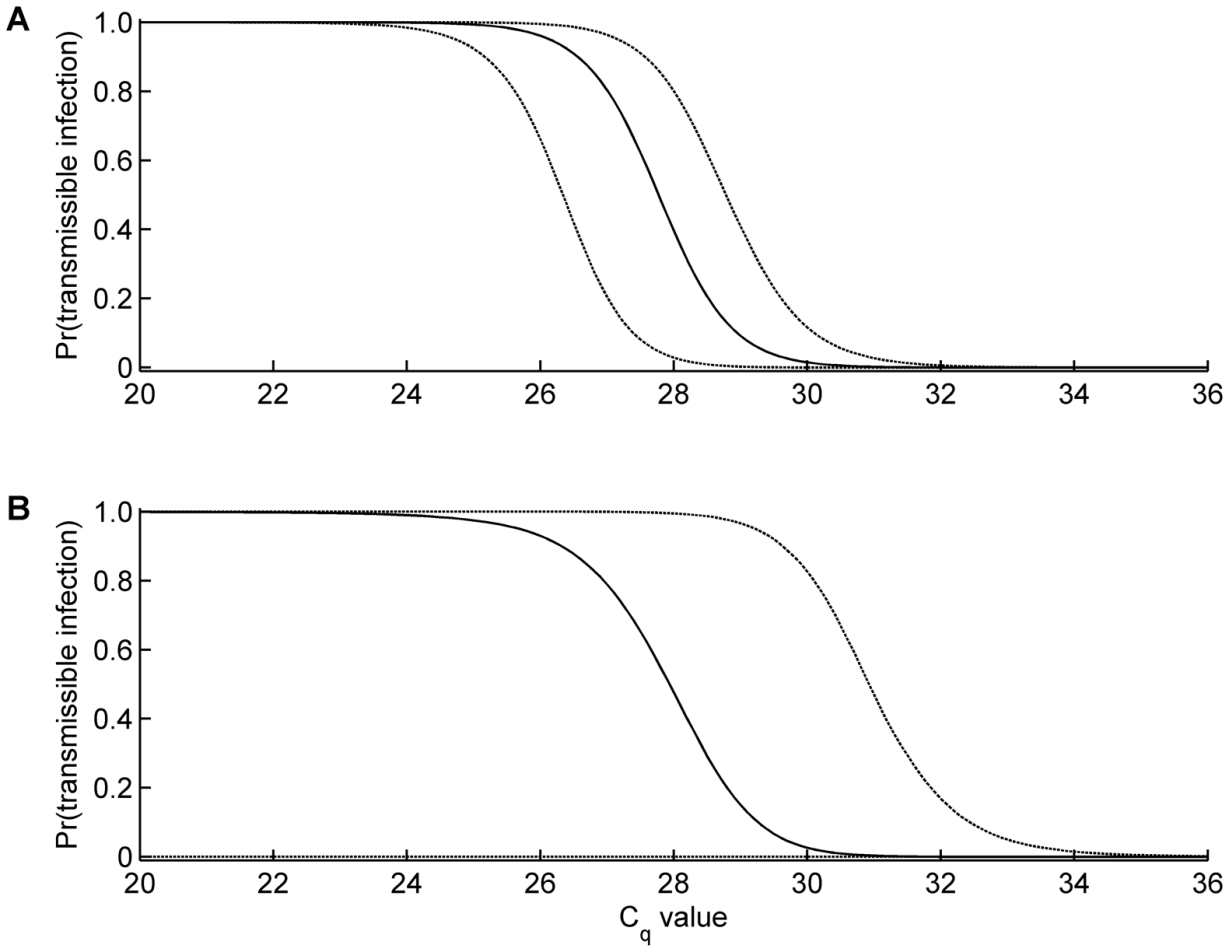
Inferred probability that *Culicoides* with a given C_q_ value has a transmissible infection (derived using **equation (2**)). Solid lines are the posterior median and the dashed lines indicate the 95% credible interval for (A) *C. sonorensis* and (B) *C. nubeculosus*.

Using the mixture model, specifically equation (2), it is possible to infer the status of a *Culicoides* based on its C_q_ value. For *C. sonorensis* a C_q_ value below 24 implies a midge will have a transmissible infection, while a C_q_ value above 32 implies a sub-transmissible infection (Figure 2A). For intermediate C_q_ values (i.e. between 24 and 32), the probability that a midge has a transmissible infection decreases from one to zero, but a particular individual could be in either class (Figure 2A). The equivalent curve for *C. nubeculosus*, indicates that midges with a C_q_ value above 34 have a sub-transmissible infection, but the equivocal results for the mixture model make it difficult to discriminate between insects with transmissible infections from those with sub-transmissible infections at lower C_q_ values (Figure 2B).

### Detection of Infectious SBV in *C. sonorensis*

Of the thirty *C. sonorensis* selected to assess the presence of infectious SBV, two had a fully disseminated infection. Infectious SBV was isolated from the head and abdomen/thorax of both individuals following inoculation onto BHK-21 cells (defined by an decrease of 7–18 C_q_ in cell monolayers between samples taken day 0 and day 4 post-inoculation). Despite passage through BHK-21 cells prior to use in *C. sonorensis*, these samples did not demonstrate a clear cytopathic effect in this cell line, demonstrating a requirement for blind passage.

## Discussion

The recovery of SBV RNA from the saliva and infectious virus from the head of orally infected *C. sonorensis* following their extrinsic incubation period in this study provides clear evidence of successful dissemination of SBV. This finding, together with previous studies of occurrence of SBV in indigenous *Culicoides* in the field [24], [25], emphasizes the increasingly important role of this genus in transmission of emerging arboviruses in northern Europe and necessitates the development of standardized techniques to infer vector competence. A key issue in making these techniques available to the wider community lies in adapting their use to frontline diagnostic technologies that are already in place in reference laboratories across this region, of which sqPCR is by far the most commonly used tool. Prior to the current SBV outbreak, investigations based solely on this technique had failed to convincingly implicate any *Culicoides* species in biological transmission beyond evidence that was already available prior to incursions taking place [38]. This resulted primarily from an under-appreciation of the importance of demonstrating full dissemination within individuals of this genus. In this study we have partially addressed this issue by directly examining levels of dissemination of SBV in laboratory reared *Culicoides* as a model to assist screening of field collected individuals involved in transmission.

SBV IT inoculation of *C. sonorensis* led to a high proportion of individuals producing saliva that contained virus RNA (eight out of ten individuals screened). These results concur with those produced for BTV [16]. These fully infected individuals provide a range of values for comparison with C_q_ values from decapitated heads of field collected *Culicoides* originating from areas of SBV transmission [24], [25]. Values provided for *C. obsoletus*, *C. scoticus* and *C. chiopterus* in the Netherlands [24] were extremely convincing and were very similar to those produced for *C. sonorensis* in the present study. In a separate study conducted in Belgium [25], far higher C_q_ values were provided from pools of heads, perhaps because, unlike the study in the Netherlands, an unoptimised homogenization step was used that could have reduced the levels of SBV RNA in samples [39].

The present study has also demonstrated that dissemination can be inferred from C_q_ values generated from whole *Culicoides*. The resulting inferred range of competence for membrane-fed individuals of both *C. sonorensis* and *C. nubeculosus* agreed closely with previous laboratory studies of these colony lines with AKAV [28]. The one study to have been published to date using pools of putatively SBV infected *Culicoides* does not convincingly demonstrate dissemination from the model produced in the current paper [40]. A major advantage of developing this alternative to the use of *Culicoides* decapitation to demonstrate arbovirus dissemination is that it is highly likely that competence rates for SBV are extremely high (which can be inferred from the fact that successful detection is currently being achieved despite using extremely small pools of individuals). In general, detection of certain other arboviruses in *Culicoides* (including BTV and African horse sickness virus) is far rarer [23], which is likely to result in decapitation becoming logistically unfeasible.

In the current study, *C*. *sonorensis* was demonstrated to be a suitable model vector species for investigations of SBV, possessing a vector competence that would be sufficient for addressing important aspects of epidemiology including determining the extrinsic incubation period and the potential for transovarial transmission. *C*. *nubeculosus,* by contrast, was largely refractory to infection, as has been found previously for a wide range of arboviruses [41]. The mixture model used to analyze the C_q_ values generated from whole insects highlights the critical difference between a susceptible and a refractory species. For the susceptible species (i.e. *C. sonorensis*) the distribution of C_q_ values is clearly bimodal, with the modes corresponding to *Culicoides* with transmissible and sub-transmissible infections (Figure 1B). By contrast, for the refractory species (i.e. *C. nubeculosus*) there is no clear bimodality and, furthermore, fewer insects have low C_q_ values (Figure 1C). Indeed, a bimodal distribution of C_q_ values could be considered as an indication that a species is a vector, with a mixture model then providing a means of quantifying its competence.

The present study has partially validated the use of an FTA® card system detection of *Culicoides*-borne arboviruses. This technique was originally developed to monitor mosquito-borne arboviruses in the field [31], and has the advantage in surveillance programs that it is extremely rapid and straightforward to deploy. While positive results were recorded for this technique from exposures to relatively large numbers of *C. sonorensis* carrying transmissible infections, the sensitivity will require further investigation as the C_q_ values produced approached the standard cut-off for the SBV assay used (≥40). Further investigation of the sensitivity of the assay under field conditions would therefore be advisable prior to use of this technique on a wide scale.

While *C. sonorensis* is a Nearctic species without direct epidemiological relevance to the northern Palaearctic, it remains the only primary arbovirus vector species of the genus worldwide that has been successfully colonized. To date attempts to colonize the major vector *Culicoides* species in Europe have failed, largely due to the inability to elicit mating under laboratory conditions and to produce equal sex ratios under the increased temperatures required to drive colony production [42]. In addition to mating at least partially in a facultative manner, *C. sonorensis* is also substantially larger, and hence more robust, than all farm-associated primary vector species in northern and southern Europe (e.g. approximate wing lengths from basal arculus to wing tip: *C. imicola* 0.9 mm; *C. sonorensis* 1.5 mm). In the case of BTV, however, this appears to translate to only slight differences in the quantity of infectious virus isolated from fully disseminated infections [43]. Bearing in mind the repeated incursions of *Culicoides*-borne viruses into the Mediterranean basin [44] and northern Europe [13], however, it is clear that there is a fundamental requirement to develop colonies of *C. imicola* and other major *Culicoides* vectors of arboviruses for both comparison with the current study and a wide range of additional areas of interest.

## Supporting Information

Figure S1 **Marginal posterior densities for parameters in a two-component mixture model, (1), for (A,C,E,G)** ***C. sonorensis*** **and (B,D,F,H)** ***C. nubeculosus*** **.** (A,B) Probability of developing a transmissible infection. (C,D) Mean and (E,F) standard deviation of the C_q_ values for *Culicoides* with a transmissible (solid line) or a sub-transmissible (dashed line) infection. (G,H) Probability of obtaining no C_q_ value for a sub-transmissible infection.
(TIF)

Figure S2 **Comparison of the observed (bars) and expected (posterior mean (circles) and 95% prediction intervals (error bars)) C_q_ values for (A)** ***C. sonorensis*** **and (B)** ***C. nubeculosus*** **infected by feeding on SBV-infected blood via a membrane.**
(TIF)

Figure S3 **Comparison of posterior predictions of the mixture model, (1), based on data for (A,B)** ***C. sonorensis*** **or (C,D)** ***C. nubeculosus*** **processed as whole insects and the results for dissected individuals.** (A,C) Predicted number of *Culicoides* with a transmissible infection following membrane feeding. The bars indicate the relative frequency and the arrow the observed number. (B,D) Predicted distribution of C_q_ values in *Culicoides* with a transmissible infection (bars) and those observed for abdomen/thorax (red symbols) or head (blue symbols) in dissected insects infected via intrathoracic inoculation (solid symbols) or membrane feeding (hollow symbols).
(TIF)

Text S1 **OpenBUGS code for implementing the two-component mixture model.**
(PDF)
