# Supplementary figures and images for: Implicating Culicoides Biting Midges as Vectors of Schmallenberg Virus Using Semi-Quantitative RT-PCR
Source: PLoS One. 2013 Mar 8;8(3):e57747. doi: 10.1371/journal.pone.0057747 (PMC3592918; doi:10.1371/journal.pone.0057747)

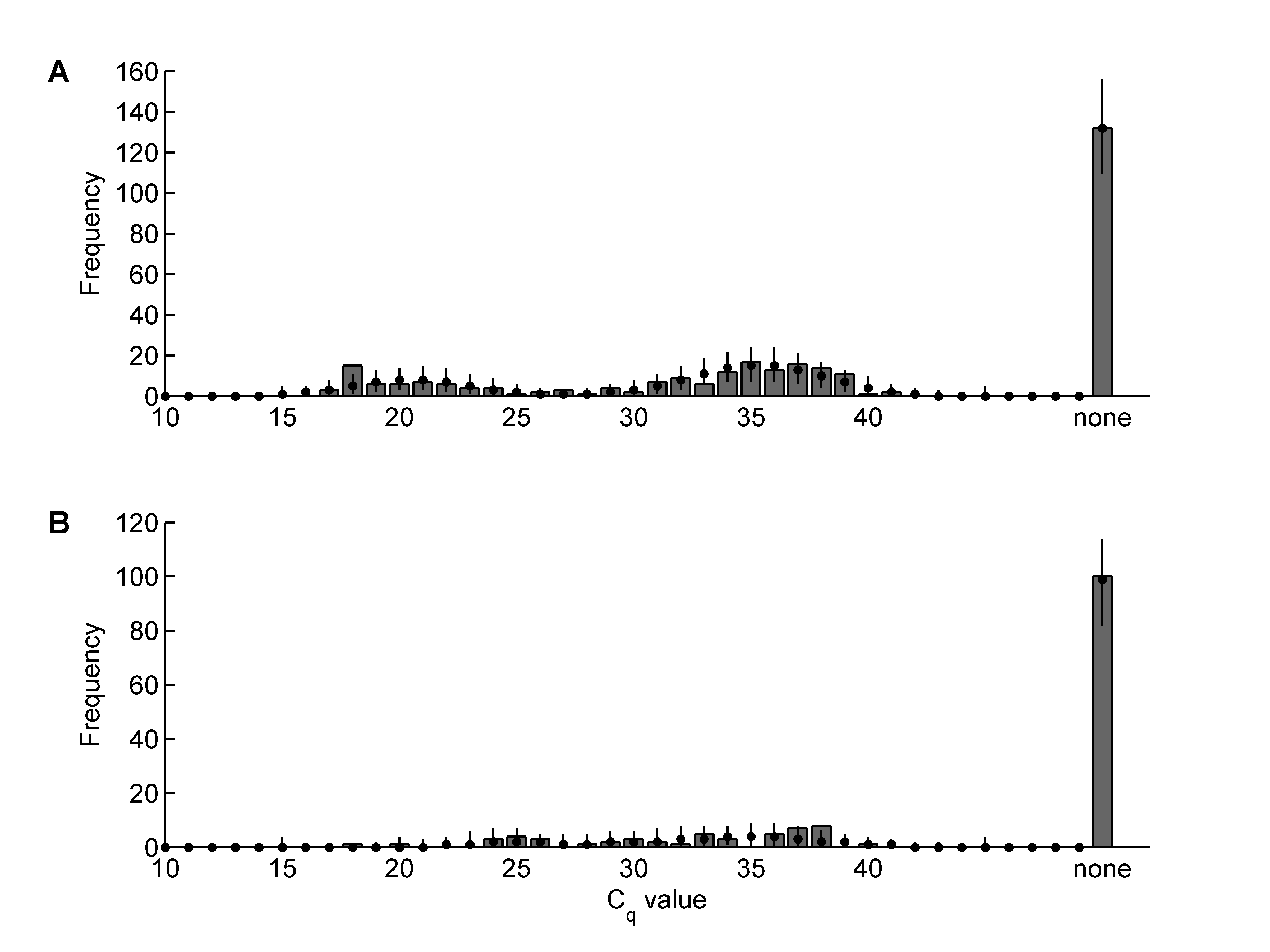

Supplement: Figure S2 — Comparison of the observed (bars) and expected (posterior mean (circles) and 95% prediction intervals (error bars)) Cq values for (A) C. sonorensis and (B) C. nubeculosus infected by feeding on SBV-infected blood via a membrane. (TIF) [file pone.0057747.s002.tif]

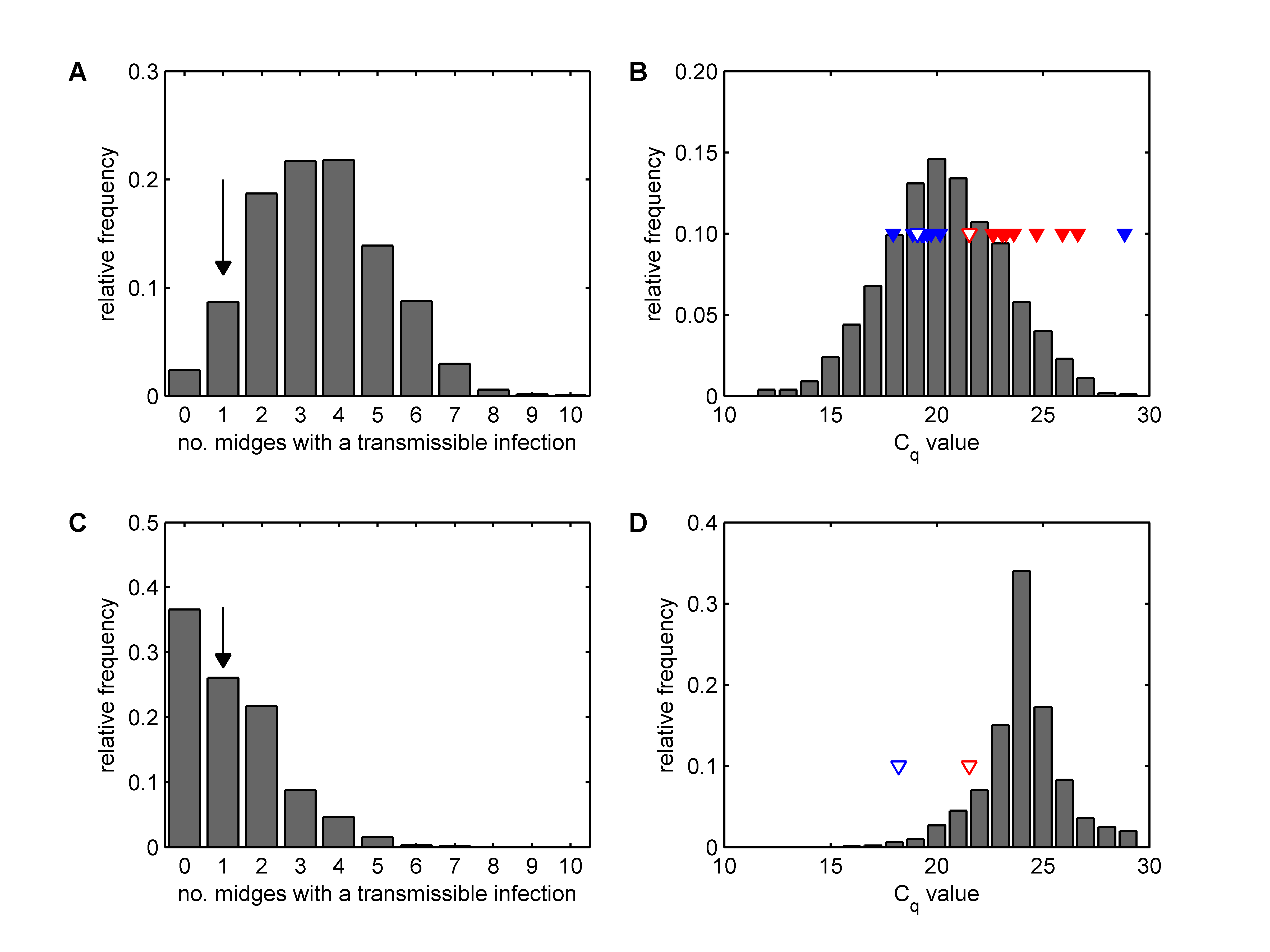

Supplement: Figure S3 — Comparison of posterior predictions of the mixture model, (1), based on data for (A,B) C. sonorensis or (C,D) C. nubeculosus processed as whole insects and the results for dissected individuals. (A,C) Predicted number of Culicoides with a transmissible infection following membrane feeding. The bars indicate the relative frequency and the arrow the observed number. (B,D) Predicted distribution of Cq values in Culicoides with a transmissible infection (bars) and those observed for abdomen/thorax (red symbols) or head (blue symbols) in dissected insects infected via intrathoracic inoculation (solid symbols) or membrane feeding (hollow symbols). (TIF) [file pone.0057747.s003.tif]
