# Supplementary material for: Implicating Culicoides Biting Midges as Vectors of Schmallenberg Virus Using Semi-Quantitative RT-PCR
Source: PLoS One. 2013 Mar 8;8(3):e57747. doi: 10.1371/journal.pone.0057747 (PMC3592918; doi:10.1371/journal.pone.0057747)
Supplement: Text S1 — OpenBUGS code for implementing the two-component mixture model. (PDF) [file pone.0057747.s004.pdf]

Text S1: **Implicating *Culicoides* biting midges as vectors of Schmallenberg virus using semi-quantitative RT-PCR**

Below is the OpenBUGS (www.openbugs.info) script used to estimate parameters in the two-component mixture model (equation (1) in the paper). The list object containing the data is assumed to consist of a vector, `Cq`, of observed C_q values and a scalar, `nobs`, giving the number of observations. Note that observations for which no C_q value was obtained are assumed to be set arbitrarily to 50.

```
model{

#==========================================================
# COMPUTE THE LOG LIKELIHOOD
# For each insect ...
  for(j in 1:nobs){

# Set a flag indicating whether or not a Cq value was observed for the
# individual
    c[j]<-step(Cq[j]-49.9)

# Compute the log PDFs for observed Cq values
    log.f0[j]<-c[j]*log(p0)+
               (1-c[j])*(log(1-p0)-log(sig[1])-
                         0.5*pow((Cq[j]-mu[1])/sig[1],2))
    log.f1[j]<-(-log(sig[2])-0.5*pow((Cq[j]-mu[2])/sig[2],2))

# Infer the infection status for each midge
    p.inf[j]<-phi*exp(log.f1[j])/((1-phi)*exp(log.f0[j])+phi*exp(log.f1[j]))
    I[j]~dbern(p.inf[j])

# Log likelihood for the Cq value (conditional on infection status)
    logL[j]<-(1-I[j])*(log(1-phi)+log.f0[j])+I[j]*(log(phi)+log.f1[j])

# Use the "zeros trick" to evaluate the likelihood for the observation
    dummy[j]<-0
    dummy[j]~dloglik(logL[j])

  }
#==========================================================

#==========================================================
# MEANS FOR THE MIXTURE COMPONENTS
# Set the means for each component of the mixture (This
# parameterisation is used to ensure identifiability of the
# parameters)
  mu[1]<-mu0+alpha
  mu[2]<-mu0
#==========================================================
```

```
#=========================================================
# PROBABILITY OF HAVING A TRANSMISSIBLE INFECTION
# Non-informative prior
  phi~dunif(0,1)
#=========================================================

#=========================================================
# PRIORS FOR C. SONORENSIS WITH A TRANSMISSIBLE INFECTION
# Non-informative priors
#  mu0~dexp(0.01)
#  sig[2]~dexp(0.01)
#=========================================================

#=========================================================
# PRIORS FOR C. NUBECULOSUS WITH A TRANSMISSIBLE INFECTION
# Informative priors based on Cq values in insects
# infected via membrane feeding sampled on day 0
  mu0~dnorm(24.2370,2.0148)
  sig[2]~dexp(1.5053)
#=========================================================

#=========================================================
# PRIORS FOR INSECTS WITH SUB-TRANSMISSIBLE INFECTIONS
# Non-informative for probability of no Cq value
  p0~dunif(0,1)

# Non-informative priors for difference in means and
# standard deviation
  alpha~dexp(0.01)
  sig[1]~dexp(0.01)
#=========================================================

}
```
